# Increase in Cardiovascular Disease Mortality in Low- and Middle-Income Countries: A Time for Action

**DOI:** 10.4314/ejhs.v35i1.1

**Published:** 2025-01

**Authors:** Elsah Tegene Asefa, Tadesse Dukessa Gemmechu, Mohammed Mecha A/fogi, Kedir Negesso Tukeni

**Affiliations:** 1 Department of Internal Medicine, Unit of cardiology, Jimma University

Cardiovascular disease (CVD) is the leading cause of death globally, accounting for nearly one-third of all deaths. The number of CVD-related deaths increased by 60%, from 12.1 million in 1990 to 20.5 million in 2021(1). This rise is primarily driven by population growth, aging, and lifestyle changes. Over 80% of CVD deaths occur in low- and middle-income countries (LMICs) faces a dual health burden, with both communicable and non-communicable diseases (NCDs) contributing to the crisis (1). The prevalence of CVD in Africa is rising, fuelled by rising rates of hypertension, smoking, and obesity (1). The continent's population diversity and healthcare infrastructure challenges complicate efforts to address CVD (2).

While improvements in the public health policies, and lifestyle changes have led to significant decline in CVD mortality in high-income countries, LMICs continue to face obstacles (1). These include low health literacy, limited access to healthcare, shortages of trained health workers, and weak healthcare infrastructure. The high cost and limited availability of CVD medications in public health facilities also pose significant challenges (3). These factors hinder prevention, diagnosis, and timely treatment of CVD in LMICs. Additionally, there is a disparity in CVD research output, with high-income countries contributing a larger share of global research despite LMICs bearing a greater CVD burden (4). This imbalance exacerbates the global CVD burden and highlights the need for equitable health interventions (1).

To tackle the CVD epidemic, a comprehensive approach is essential. Strengthening healthcare systems, implementing effective public health policies, and promoting healthy lifestyles are key priorities (1). Global initiatives like the World Health Organization's Global Heart Initiative aim to reduce CVD risk factors through evidence-based interventions. Such initiatives are encouraging and can support CVD researches and interventions, particularly in LMICs. A sustained, collaborative commitment across nations is necessary to reduce the global CVD burden and improve cardiovascular health. Efforts to lower CVD mortality must also consider the socio-economic and cultural contexts of the affected communities (2). Addressing these gaps through policy intervention and increased public financing is critical for preventing CVD and reducing mortality in LMICs.
